# Sparse Transform and Compressed Sensing Methods to Improve Efficiency and Quality in Magnetic Resonance Medical Imaging

**DOI:** 10.3390/s25165137

**Published:** 2025-08-19

**Authors:** Santiago Villota, Esteban Inga

**Affiliations:** 1 Biomedical Engineering Program, Universidad Politécnica Salesiana, Quito EC170525, Ecuador; svillota@est.ups.edu.ec; 2 Master in ICT for Education, Smart Grid Research Group (GIREI), Universidad Politécnica Salesiana, Quito EC170525, Ecuador

**Keywords:** compressed sensing, magnetic resonance imaging, basis pursuit, signal reconstruction, optimization, PSNR, RMSE

## Abstract

This paper explores the application of transform-domain sparsification and compressed sensing (CS) techniques to improve the efficiency and quality of magnetic resonance imaging (MRI). We implement and evaluate three sparsifying methods—discrete wavelet transform (DWT), fast Fourier transform (FFT), and discrete cosine transform (DCT)—which are used to simulate subsampled reconstruction via inverse transforms. Additionally, one accurate CS reconstruction algorithm, basis pursuit (BP), using the L_1_-MAGIC toolbox, is implemented as a benchmark based on convex optimization with L_1_-norm minimization. Emphasis is placed on basis pursuit (BP), which satisfies the formal requirements of CS theory, including incoherent sampling and sparse recovery via nonlinear reconstruction. Each method is assessed in MATLAB R2024b using standardized DICOM images and varying sampling rates. The evaluation metrics include peak signal-to-noise ratio (PSNR), root mean square error (RMSE), structural similarity index measure (SSIM), execution time, memory usage, and compression efficiency. The results show that although discrete cosine transform (DCT) outperforms the others under simulation in terms of PSNR and SSIM, it is inconsistent with the physics of MRI acquisition. Conversely, basis pursuit (BP) offers a theoretically grounded reconstruction approach with acceptable accuracy and clinical relevance. Despite the limitations of a controlled experimental setup, this study establishes a reproducible benchmarking framework and highlights the trade-offs between the quality of transform-based reconstruction and computational complexity. Future work will extend this study by incorporating clinically validated CS algorithms with L_0_ and nonconvex Lp (0 < *p* < 1) regularization to align with state-of-the-art MRI reconstruction practices.

## 1. Introduction

Optimization in the reconstruction of medical image data addresses the modern problem of long processing times, particularly in magnetic resonance imaging (MRI). The concept of compressed sensing (CS) emerged in the mid-2000s as a transformative theory in signal processing, fundamentally changing how data could be acquired and reconstructed. The foundational works of Donoho [1] and Candes, Romberg, and Tao [2] demonstrated that it is possible to reconstruct signals from far fewer samples than traditionally required, provided the signals are sparse in some domain and the sampling is incoherent. One of the first real-world applications of CS was in medical imaging, notably by Lustig et al. [3], who showed how CS can be effectively applied to accelerate magnetic resonance imaging (MRI). Since then, CS has been used across a wide range of domains, including biomedical imaging, radar, wireless communication, remote sensing, and more [4].

While advanced reconstruction methods—most notably the Nesterov-based first-order sparse recovery algorithm (NESTA), developed by Becker, Bobin, and Candès [5]—have been widely used for sparse signal recovery, this study focuses on how sparse transforms—such as the Discrete Fourier Transform (DFT), the Discrete Cosine Transform (DCT), and the Discrete Wavelet Transform (DWT)—can be applied for subsampled reconstruction within compressed sensing frameworks. In particular, Compressed Sensing (CS) via L_1_-norm minimization enables effective signal recovery through optimization algorithms such as Basis Pursuit, which help reduce computational costs and reconstruction time [6].

MRI scans often require long scan times, approximately 30 to 40 min [7], which can be uncomfortable for patients and limit the ability of medical centers to handle large numbers of patients. Additionally, the MRI image acquisition process generates large volumes of data, increasing storage and processing costs. This process, called “spatial coding”, involves a matrix array in “K-space”. If not filled with sufficient information between slices and necessary sampling, it presents unwanted aliasing artifacts [8,9].

Thanks to CS, line-by-line sampling of K-space is avoided and reduced to random subsampling, as long as the information is encoded in samples and not directly in pixels [6,10,11]. The CS process can be grouped into three sections according to Mishra Ishani [12]: First, sparse representation, where a signal or dataset is represented as a linear combination of essential elements of a “dictionary”, with most unchosen coefficients being zero [13]; second, sampling, where relevant data are collected using domains or dictionaries such as wavelet, enhancing the sparse values for efficient selection [14]; and third, reconstruction algorithms, which may vary according to their application, including basis pursuit (BP) and orthogonal matching pursuit (OMP) [15,16,17].

When dealing with fast MRI reconstruction, three critical variables are sparsity, incoherence, and nonlinear reconstruction. Sparsity refers to the ability to compress images by applying a threshold to retain only the necessary coefficients and discard the less important ones, similar to JPEG compression. Incoherence involves pseudo-random undersampling of k-space to prevent the production of aliasing artifacts, while iterative algorithms perform nonlinear reconstruction [18].

The correct choice of reconstruction algorithm depends on several factors, including the type of data being processed, the signal’s characteristics, and computational constraints. For example, some algorithms may be more suitable for signals with specific sparsity properties, while others may be more efficient in terms of computational time or memory usage. In some applications, minimizing the reconstruction error is critical to ensure diagnostic accuracy, while in others, reducing the reconstruction time to enable real-time processing is more critical [19].

**Figure 1:**
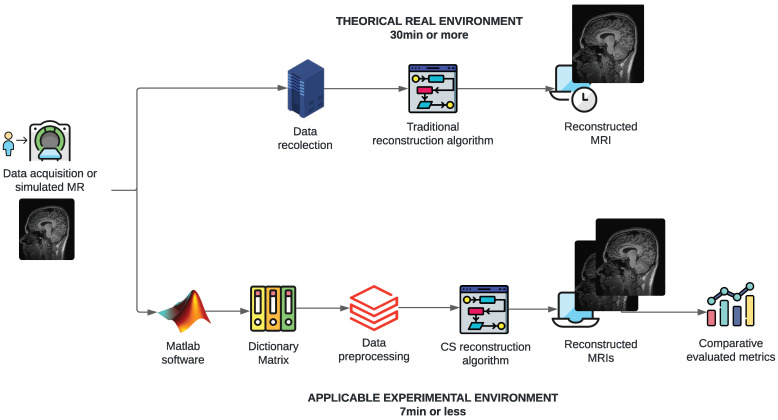
Conceptual figure illustrating the problem and proposed solution.

Figure 1 illustrates the problem and proposed solution conceptually. The figure illustrates the steps involved, including the use of MATLAB for preprocessing, the application of CS algorithms, and the reconstruction process. It provides a visual overview of the approach taken to address the long processing times of MRI scans. The figure shows the theoretical–methodological process, starting with a signal or sample image obtained from a publicly accessible medical database for research purposes. It compares the process through two timelines, highlighting the creation and application of the CS algorithm and indicating the proposed times of 7 min or less compared to the 30 min that MR studies commonly take.

Given prior knowledge of the sparse nature and complexity of MRI signals, this study proposes an experimental implementation in the MATLAB software that utilizes the fundamentals of compressed sensing (CS) to develop essential methods for future innovative techniques. Metrics such as the root mean square error (RMSE), peak signal-to-noise ratio (PSNR), and structural similarity index measure (SSIM) are used to evaluate the quality of reconstructed images. Tools like the L_1_-Magic toolbox are implemented in MATLAB to utilize CS algorithms [20]. Threshold denoising techniques and suboptimal sampling are critical components in this process.

The study commences with a comprehensive examination of methods utilizing CS techniques. Among these, only the fourth algorithm follows the formal definition of compressed sensing by solving an L_1_-norm minimization problem. The remaining algorithms are based on sparse-domain subsampling followed by inverse transforms and serve as computational baselines rather than accurate CS methods. The results are collected through different sampling rates and a detailed analysis. Finally, the conclusions from the experimental study are presented, and the relevance and implications of the methods used are discussed.

This study does not propose new reconstruction algorithms; instead, it presents a novel and reproducible experimental framework for systematically evaluating established sparse transforms in compressed sensing for MRI. The comparative analysis is carried out under unified simulation conditions, using publicly available DICOM images and consistent sampling rates, allowing the benchmarking of discrete wavelet transform (DWT), fast Fourier transform (FFT), discrete cosine transform (DCT), and basis pursuit (BP) using a coherent set of quantitative metrics (PSNR, RMSE, SSIM, memory usage, and execution time). This level of integration and normalization across transform domains has not been comprehensively reported in prior studies; it will provide practical insights into the trade-offs between reconstruction quality and computational complexity in MRI settings. The results provide a foundation for future hybrid systems and facilitate replication in subsequent research.

## 2. Related Works

Compressed sensing (CS) has proven to be a versatile tool in several domains, particularly in reconstructing sparse signals from fewer measurements than traditional methods. In the field of wireless communications, Azarnia et al. [21] introduced a novel PAPR reduction method in orthogonal frequency-division multiplexing (OFDM) systems, applying CS on the transmitter side and orthogonal matching pursuit (OMP) at the receiver. These techniques significantly improved signal integrity and compression.

The application of CS in medical imaging, and particularly MRI, has evolved in tandem with algorithmic innovations. Canon Medical Systems developed Compressed SPEEDER, which integrates CS with parallel imaging (PI) to enhance both image quality and acquisition speed. Their implementation within PACS systems showed superior outcomes compared to CS-alone reconstructions. Similarly, Cao et al. [22] proposed a new CS-MRI model incorporating dictionary learning and manifold structure regularization. Their approach relied on a tight frame constraint, which enabled a more practical and sparse representation, leading to improved reconstruction fidelity.

Transform-domain CS approaches remain central in this field. For instance, the Daubechies wavelet transform, as utilized in Dwork’s study [23], exploits affine transformations to improve sparsity before reconstruction, ultimately framing the problem as a basis pursuit denoising (BPDN) optimization. This approach was shown to yield lower reconstruction error bounds across both magnetic resonance and optical imaging tasks. Complementing this, Abramova et al. [19] explored DCT-based lossy compression by predicting quantization-induced mean square errors and analyzing spectral distortion characteristics, demonstrating DCT’s efficiency in encoding smooth regions.

FFT has also been integrated into hybrid frameworks. Wang et al. [24] proposed a visually meaningful dual-image encryption algorithm combining FFT, chaotic systems, and parallel CS. Their use of a Hadamard matrix and wavelet-transformed images enabled encrypted reconstruction with preserved resolution and robustness.

The recent literature also reflects increasing integration of CS with neural methods. Hosny et al. [25] highlight how matching pursuit algorithms (BP, MP, OMP) are now hybridized with deep learning structures to further enhance reconstruction accuracy and generalizability. Emerging frameworks have begun incorporating neural priors and regularization terms learned from data, pushing the boundary of undersampling limits.

In terms of algorithmic acceleration, Cai and his team proposed a domain-based fast retrieval method comprising Network-based Projected Gradient Descent (NPGD) and Denoiser-based Proximal Gradient Descent (DPGD), achieving reduced computation time and improved reconstruction quality in high-resolution scenarios.

Altogether, these works demonstrate that while CS has matured substantially over the past decade, opportunities remain for comparative evaluations across different transform domains. The present study contributes by implementing a unified framework to evaluate DWT, FFT, DCT, and BP under fixed sampling conditions and standardized metrics, offering insight into practical trade-offs between reconstruction quality, sparsity, and computational cost.

In addition to classical reconstruction techniques, deep learning approaches have recently demonstrated strong potential for accelerating and improving the quality of MRI reconstructions. Convolutional neural networks, recurrent models, and, more recently, generative and vision–language architectures have been applied to reduce sampling rates and reconstruction time while preserving diagnostic quality. One notable example is the work in [26], which introduces general-purpose vision–language models for medical image analysis, including MRI, and outlines a unified framework for combining semantic information and image features to support robust reconstruction. Although these techniques are beyond the scope of the current implementation, they represent a promising research direction for future extensions of this work.

Despite significant progress in compressed sensing theory and applications, the present study evaluates only one CS algorithm—basis pursuit—using the L_1_-MAGIC toolbox, which was initially developed during the early stages of CS research. However, several more advanced and clinically validated CS frameworks now exist. For instance, SPARSE-MRI [3], SPARSE-SENSE [27], L_1_-SPIRiT [28], and k-t SPARSE-SENSE [29] have been applied successfully in anatomical, parallel, and dynamic MRI. Some of these methods have received FDA approval for clinical use [30,31,32], evidencing the maturity of CS technologies for real-world deployment. These methods outperform early L_1_-based reconstructions in robustness and speed, especially when implemented with hardware acceleration or adaptive priors. Future versions of this work should incorporate such algorithms to enable a more representative and up-to-date comparison.

## 3. Problem Formulation and Methodology

Magnetic resonance imaging (MRI) plays a vital role in diagnosing musculoskeletal, neurological, and cardiovascular conditions, where the identification of subtle pathologies demands high-resolution images. However, complete acquisition of k-space data leads to prolonged scan times, limiting the frequency and availability of diagnostic studies. This study aims to improve acquisition speed and reduce computational burden by applying compressed sensing (CS) algorithms for MRI reconstruction. Specifically, the focus is on comparing the efficiency of different sparsifying transforms and optimization techniques to achieve accurate image reconstruction from undersampled data.

In MRI, the acquisition process is modeled as y=F(x), where *F* denotes the Fourier transform of the spatial-domain image *x*. Consequently, reconstruction involves solving this inverse problem.

**Figure 2:**
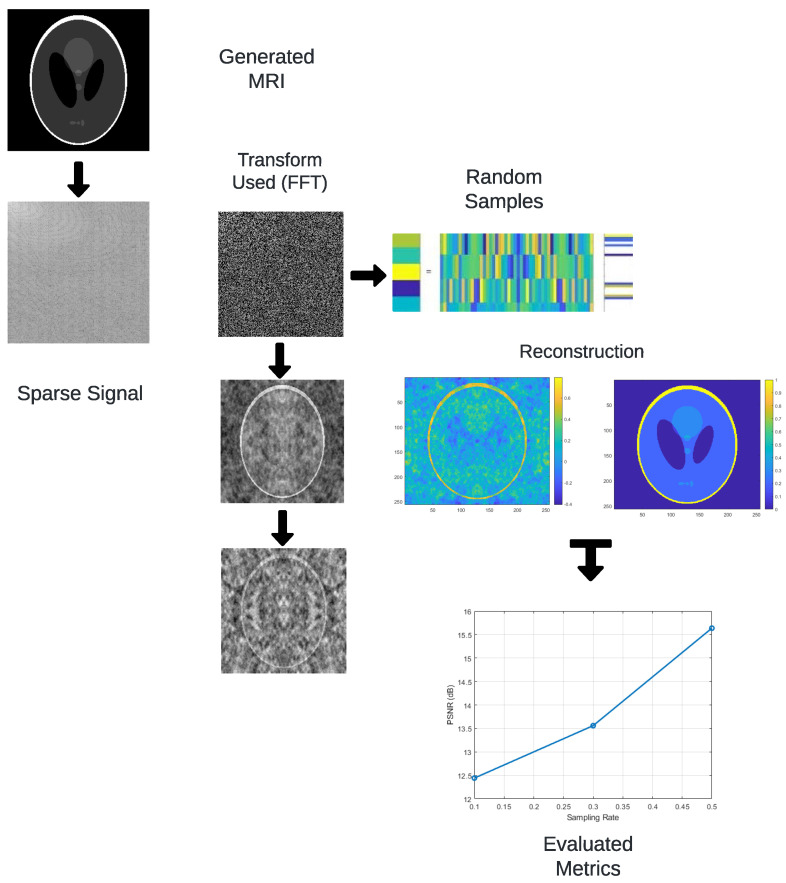
Workflow of the proposed methodology. Although simplified, this representation reflects the transform-domain sparsification and optimization-based reconstruction pipeline employed in the experiments.

Figure 2 summarizes the methodological workflow. The process begins by transforming the spatial-domain image using the FFT, DCT, or DWT. A random subset of coefficients is sampled to simulate undersampled acquisition. The reconstruction is performed via inverse transforms or L_1_-based optimization (basis pursuit), and quality is assessed using the RMSE, PSNR, and SSIM metrics. It begins with generating an image representing the original signal. The FFT is applied to convert the image from the spatial to the frequency domain, a step that can also be achieved using other transforms, such as DCT or DWT. A random sampling of the transformed coefficients is then performed, reducing the data needed for reconstruction. The signal is reconstructed in the spatial domain by applying the inverse of the used transforms, utilizing optimization algorithms like basis pursuit. Finally, reconstruction quality metrics such as RMSE, PSNR, and SSIM are evaluated through detailed comparisons.

Although Figure 2 is presented in a simplified visual format to prioritize clarity, it is intended to convey the essential stages of the proposed methodology. The schematic illustrates the overall process flow, from transform-domain sparsification to random sampling and reconstruction. While not all connections between steps are explicitly shown as arrows, the textual explanation in this section provides a detailed account of the data dependencies and algorithmic stages involved. The diagram serves as a conceptual aid, and its simplified layout aligns with the descriptive depth provided in the accompanying narrative.

The study was conducted on a computer with the following specifications: Intel(R) Xeon(R) CPU E5-2683 v4 @ 2.10 GHz (2 processors), 64.0 GB of installed RAM, and a 64-bit operating system with an x64-based processor. These specifications ensure optimal performance for executing the algorithms, enabling efficient processing and handling of large datasets.

Table 1 summarizes the notation and variables used across all mathematical formulations and algorithmic implementations discussed in this work.

### 3.1. Principles of CS Application in MRI

CS theory relies on three core principles: sparsity, incoherence, and nonlinear reconstruction. In MRI, sparsity is achieved by representing the image in a transform domain (e.g., wavelet), incoherence is enforced via random undersampling in k-space, and reconstruction is performed by solving an optimization problem that minimizes a norm (typically L_1_) under fidelity constraints. Detailed explanations of these principles are found in reviews such as [4,33].

Compressed sensing (CS) theory posits that if a signal is sparse or compressible in some domain, it can be reconstructed from a small number of incoherent linear measurements, significantly below the Nyquist rate. In mathematical terms, the problem is posed as solving y=Φx, where *x* is the signal (or image), Φ is a sensing matrix, and *y* is the observed measurement. The reconstruction involves solving

$$\min_{x}{∥x∥}_{1}\;\mathrm{subject}\;\mathrm{to}\;y=\Phi x$$

In MRI, this translates to undersampling the k-space data and reconstructing the image using sparsity priors. The measurement operator *F* is typically the Fourier transform, so the MRI model becomes y=F(x). The goal is to recover *x* from undersampled *y* by exploiting sparsity in a transform domain (e.g., wavelet or DCT) and solving an optimization problem.

The fundamental principles of CS in MRI are detailed in surveys such as Feng et al. [34], and the practical importance of sparsifying transforms and incoherent sampling is outlined in recent evaluations like Konovalov [4].

### 3.2. Description of Reconstruction Algorithms

#### 3.2.1. Algorithm 1: Discrete Wavelet Transform (DWT)

Algorithm 1 uses suboptimal sampling with the Haar wavelet transform for image compression and reconstruction. Key steps include ‘dicomread’ to read the image, ‘im2double’ and ‘mat2gray’ for normalization, ‘wavedec2’ for wavelet decomposition, ‘randsample’ to select coefficients, and ‘waverec2’ for image reconstruction. Additionally, ‘imshow’ and ‘title’ display the images.
**Algorithm 1** Processing using Wavelet1:**function** processImage(image)2:    A←dicomread(image)3:    A←im2double(A)4:    A←mat2gray(A)5:    signal←double(A)6:    samplingRate←0.57:    $\left[\mathrm{coeffs},\mathrm{bookkeeping}\right]\leftarrow \mathrm{wavedec}2\left(signal,1{,}^{\prime }haar^{\prime }\right)$8:    M←length(coeffs)9:    N←round(M×samplingRate)10:    indices←randsample(1:M,N)11:    y←coeffs(indices)12:    recoveredCoeffs←zeros(1,M)13:    **for** i∈indices **do**14:        recoveredCoeffs[i]←y[i]15:    **end for**16:    $recoveredSignal\leftarrow \mathrm{waverec}2(recoveredCoeffs,\mathrm{bookkeeping}{,}^{\prime }haar^{\prime })$17:$\;\;\;\;\mathrm{for}\;(img,titleText)\in [\left(signal{,}^{\prime }OriginalImage^{\prime }\right),\;\left(recoveredSignal,[’\mathrm{Recovered}\;\mathrm{Image}\;\mathrm{with}\;\mathrm{Sampling}\;\mathrm{Rate}:\;’,\mathrm{num}2\mathrm{str}\left(\mathrm{samplingRate}\right)])\right]\;\mathrm{do}$18:        imshow(img, [])19:        title(titleText)20:    **end for**21:    results←computeMetrics(signal,recoveredSignal,y)22:    **return** results23:**end function**24:**function** computeMetrics(original,recovered,sampledCoeffs)25:    time_elapsed←toc26:    memory_used←memory27:    compression←norm(sampledCoeffs)/norm(original)28:    mse←immse(original,recovered)29:    $rmse\leftarrow \sqrt{mse}$30:    psnr←psnr(recovered,original)31:    ssim←ssim(recovered,original)32:    return {time_elapsed,memory_used,compression,mse,rmse,psnr,ssim}33:**end function**

#### 3.2.2. Algorithm 2: Fast Fourier Transform (FFT)

Similarly, Algorithm 2 employs FFT for image compression and reconstruction, utilizing ‘fft2’ to transform the image to the frequency domain, ‘randsample’ to select frequency coefficients, and ‘ifft2’ for inverse transformation. ‘real’ is used to extract the fundamental part of the reconstructed image.
**Algorithm 2** Processing using FFT1:**function** compressionFFT(image)2:    A←dicomread(image)3:    A←im2double(A)4:    A←mat2gray(A)5:    signal←double(A)6:    samplingRate←0.57:    coeffs←fft2(signal)8:    M←numel(coeffs)9:    N←round(M×samplingRate)10:    indices←randsample(1:M,N)11:    y←coeffs(indices)12:    recoveredCoeffs←zeros(size(coeffs))13:    **for** i∈indices **do**14:        recoveredCoeffs[i]←y[i]15:    **end for**16:    recoveredSignal←real(ifft2(recoveredCoeffs))17:    results←calculateMetrics(signal,recoveredsignal,y)18:    **return** results19:**end function**20:**function** calculateMetrics(original,recovered,sampledCoeffs)21:    time_elapsed←toc22:    memory_used←memory23:    compression←norm(sampledCoeffs)/norm(original)24:    mse←immse(original,recovered)25:    $rmse\leftarrow \sqrt{mse}$26:    psnr←psnr(recovered,original)27:    ssim←ssim(recovered,original)28:    return {time_elapsed,memory_used,compression,mse,rmse,psnr,ssim}29:**end function**

#### 3.2.3. Algorithm 3: Discrete Cosine Transform (DCT)

Algorithm 3 utilizes the Discrete Cosine Transform (DCT) for image compression and reconstruction. It uses ‘dct2’, ‘sort’ to select significant coefficients, and ‘idct2’ for image reconstruction. ‘zeros’ is used to initialize arrays for chosen coefficients.

#### 3.2.4. Algorithm 4: Basis Pursuit via L_1_-MAGIC

Algorithm 4 applies Compressed Sensing through Basis Pursuit using the L_1_-MAGIC MATLAB toolbox [20]. Specifically, it solves an optimization problem of the form:

$$\min_{s}{∥s∥}_{1}\;\mathrm{subject}\;\mathrm{to}\;\Theta s=y$$

where *s* is the sparse coefficient vector, *y* is the measurement vector, and $\Theta =\Phi \Psi^{-1}$ is the effective sensing matrix formed by the random projection matrix Φ and the inverse sparsifying transform $\Psi^{-1}$ (in this case, the inverse DCT).

The function l1eq_pd is a primal-dual interior-point solver for convex optimization problems. It efficiently minimizes the L_1_ norm of the signal representation under equality constraints. This solver iteratively finds a solution by jointly updating primal and dual variables while maintaining feasibility and optimality conditions. The method is suitable for problems in which the signal is sparse in some domain and the measurement matrix satisfies the restricted isometry property (RIP).

This approach faithfully implements one of the earliest and most widely cited forms of CS reconstruction. However, it should be noted that L_1_-MAGIC has been surpassed by more modern algorithms in terms of speed and robustness, especially in clinical MRI settings. Nonetheless, it serves as a valid benchmark for evaluating the basic performance of L_1_-based sparse recovery.
**Algorithm 3** Processing using DCT1:**function** compressionDCT(image,samplingRate)2:    A←dicomread(image)3:    A←im2double(A)4:    A←mat2gray(A)5:    x←double(A)6:    coeffs2D←dct2(x)7:    N←floor(samplingRate×numel(coeffs2D))8:    $\left[sortedCoeffs,indices\right]\leftarrow \mathrm{sort}(\mathrm{abs}\left(coeffs2D\left(:\right)\right){,}^{\prime }descend^{\prime })$9:    retainedIndices←indices(1:N)10:    y←zeros(size(coeffs2D))11:    **for** i∈retainedIndices **do**12:        y[i]←coeffs2D[i]13:    **end for**14:    x1←idct2(y)15:    results←calculateMetrics(x,x1,y)16:    **return** results17:**end function**18:**function** calculateMetrics(original,recovered,sampledCoeffs)19:    time_elapsed←toc20:    memory_used←memory21:    compression←norm(sampledCoeffs)/norm(original)22:    mse←immse(original,recovered)23:    $rmse\leftarrow \sqrt{mse}$24:    psnr←psnr(recovered,original)25:    ssim←ssim(recovered,original)26:    **return** {time_elapsed,memory_used,compression,mse,rmse,psnr,ssim}27:**end function**

Among the four implementations, only Algorithm 2 (FFT-based reconstruction) and Algorithm 4 (Basis Pursuit via L_1_-MAGIC, DCT domain) are theoretically consistent with the MRI measurement model, since in real MRI acquisition the measured signal corresponds to Fourier samples of the desired image. In contrast, Algorithm 1 (DWT) and Algorithm 3 (DCT) rely on transform-domain subsampling and inverse transforms that do not replicate the k-space acquisition process. These two methods are therefore not suitable for clinical MRI reconstruction; their inclusion in this study is purely experimental, serving as benchmarks to explore the behavior of sparsifying transforms under controlled undersampling conditions.

In order to provide the mathematical background of the transforms used in these algorithms, the following subsection details their theoretical formulation and properties.

#### 3.2.5. Matrix Dictionary and Discrete Transforms

The FFT is an algorithm for calculating the Discrete Fourier Transform (DFT) quickly and efficiently. The DFT transforms a signal from the temporal or spatial domain to the frequency domain. In the context of processing, this process converts the gray-level distribution of an image into a frequency distribution. An image that is a discrete sequence of points in the spatial domain is represented in the frequency domain by the following expression:

(1) $$F\left(u,v\right)=\sum_{x=0}^{M-1}\sum_{y=0}^{N-1}f\left(x,y\right)\cdot e^{-i2\pi \left(\frac{ux}{M}+\frac{vy}{N}\right)}$$

Here, *x* and *y* are discrete variables in the spatial domain, while *u* and *v* are discrete variables in the frequency domain. The physical interpretation of the FFT in image processing is the conversion of the distribution of gray levels in an image into a frequency distribution [24].
**Algorithm 4** Compressed Sensing using Basis Pursuit1:**function** compressionBP(image, samplingRate)2:    A←dicomread(image)3:    A←im2double(A)4:    A←mat2gray(A)5:    A←imresize(A,[128,128])6:    A(A<0.1)←07:    A←(A−min(A(:)))/(max(A(:))−min(A(:)))8:    signal←A(:)9:    m←round(length(signal)×samplingRate)10:    $Phi\leftarrow \mathrm{randn}\left(m,\mathrm{length}\left(signal\right)\right)/\sqrt{m}$11:    y←Phi×signal12:    Phi←Phi+eye(size(Phi))×1e−613:    Theta←Phi×idct(eye(length(signal)))14:    $s1\leftarrow l1\mathrm{eq}\_\mathrm{pd}(\mathrm{zeros}\left(\mathrm{length}\left(signal\right),1\right),Theta,Theta^{\prime },y,1e-6,26)$15:    recoveredSignal←idct(s1)16:    recoveredSignal←reshape(recoveredSignal,size(A))17:    recoveredSignal(recoveredSignal<0.1)←018:    results←calculateMetrics(signal,recoveredsignal,y)19:    **return** results20:**end function**21:**function** calculateMetrics(original,recovered,sampledCoeffs)22:    time_elapsed←toc23:    memory_used←memory24:    compression←∥sampledCoeffs∥/∥original∥25:    mse←immse(original,recovered)26:    $rmse\leftarrow \sqrt{mse}$27:    psnr←psnr(recovered,original)28:    ssim←ssim(recovered,original)29:    **return** {time_elapsed,memory_used,compression,mse,rmse,psnr,ssim}30:**end function**

The Wavelet transform dictionary is a recurrent method that replaces the Fourier transform as the classical way for data reconstruction and analysis. It can work in the frequency-time domain, ensuring its isotropy, making it suitable for MRI processing [35,36]. Chao shows that the base formula of a Wavelet is expressed as follows [37]:

(2) $$\psi_{u,s}\left(t\right)=\psi \left(\frac{t-u}{s}\right)$$

In this formula, $\psi_{u,s}\left(t\right)$ represents a family of wavelet functions generated from a mother function ψ(t) by shifting *u* and scaling *s*. This property enables the analysis of the signal at various resolutions.

It is important to note that the use of L_1_-norm minimization in Basis Pursuit leads to a convex optimization problem, which guarantees the existence of a unique global minimum under appropriate conditions, such as the Restricted Isometry Property (RIP) being satisfied by the sensing matrix Φ [38]. Therefore, issues related to local minima are avoided, and the reconstruction is robust to noise and undersampling, provided that the sparsity prior holds. However, in practical implementations, robustness may still depend on numerical stability and conditioning of the inverse problem, which are influenced by the sampling strategy and signal structure.

### 3.3. Quality Assessment Metrics

#### 3.3.1. Stability Constants

The constants $C_{1}$ and $C_{2}$ are defined to stabilize the division with weak denominators. Typically, they are set as:

(3) $$C_{1}={\left(K_{1}L\right)}^{2},\;C_{2}={\left(K_{2}L\right)}^{2},$$

where *L* is the dynamic range of the pixel values (e.g., 255 for 8-bit grayscale images), and $K_{1}=0.01,\;K_{2}=0.03$ are standard bias parameters.

Together, these metrics provide a comprehensive evaluation framework to compare transform-based reconstruction methods across accuracy, fidelity, and computational cost.

#### 3.3.2. Structural Similarity Index Measure (SSIM)

The Structural Similarity Index Measure (SSIM) measures similarity between two images by comparing structural, luminance, and contrast properties. It is calculated as:

(4) $$\mathrm{SSIM}\left(x,x_{1}\right)=\frac{\left(2\mu_{x}\mu_{x_{1}}+C_{1}\right)\left(2\sigma_{xx_{1}}+C_{2}\right)}{\left(\mu_{x}^{2}+\mu_{x_{1}}^{2}+C_{1}\right)\left(\sigma_{x}^{2}+\sigma_{x_{1}}^{2}+C_{2}\right)},$$

where $\mu_{*}$ and $\sigma_{*}^{2}$ denote means and variances of the image windows, and $\sigma_{xx_{1}}$ their covariance.

#### 3.3.3. Norms of CS

The CS problem is effectively addressed using a sparse regularization technique, specifically the *L*_1_ norm. This technique poses potential alternatives and patterns for resolution in the sparse domain [39], based on collecting a fraction of the available K-space data [40].

In the context of image deconvolution, the *L*_1_ norm facilitates the recovery of fine details and unique cellular structures by using fading masks that effectively remove noise. Due to its convex nature [41], this represents a nonlinear problem that nonlinear optimization algorithms can solve [42].

There are various applications of the *L*_1_ norm; however, in this specific project, we will utilize *L*_1_-MAGIC in MATLAB, which encompasses several algorithms and routines for solving convex and compressive sensing problems.

The Discrete Cosine Transform (DCT) dictionary is a technique that decomposes a signal into cosine functions of varying frequencies, similar to the Fourier Transform. It can highly concentrate energy in the frequency domain, which is beneficial for sparse transformation in MRI [43]. Even in the filtering context, the DCT can be used with the Top-hat filter for noise removal [44]. Xu Zhang accurately describes the mathematical concept behind the DCT [45]; its representation is as follows:

(5) $$D\left(u,v\right)=\frac{2}{\sqrt{m\times n}}\sum_{i=1}^{n}\sum_{j=1}^{m}A\left(i,j\right)\cos\left(\frac{(2i-1)(u-1)\pi }{2n}\right)\cos\left(\frac{(2j-1)(v-1)\pi }{2m}\right)$$

In this equation, D(u,v) represents the Discrete Cosine Transform (DCT) coefficients for a matrix A(i,j) of size m×n, where *i* and *j* are the indices of the matrix elements. The DCT allows for a compact and efficient representation of the original signal.

Thus, both Equations (2) and (5) express the obtaining of Wavelets and the DCT coefficients, respectively. The symbols and their meanings can be found in Table 1.

(6) Θ=DCT(C)=Ψ(C)

The selection of the appropriate transform in MRI reconstruction is not arbitrary but depends on the characteristics of the medical image and the objectives of the reconstruction process. The Discrete Wavelet Transform (DWT) is well-suited for preserving edges and anatomical boundaries, as it provides a multiscale analysis and is capable of capturing both frequency and spatial information with sound localization. It is particularly advantageous in MRI, where soft tissue contrasts and delicate structures are relevant. The Fast Fourier Transform (FFT), by contrast, is inherently linked to the frequency-domain data acquisition used in MRI (k-space). Its direct relationship with MRI hardware and sampling trajectories makes it a natural choice for rapid encoding and reconstruction, although it is more sensitive to undersampling artifacts when incoherence is not ensured. Meanwhile, the Discrete Cosine Transform (DCT) exhibits strong energy compaction properties, making it highly efficient for representing smooth intensity variations in homogeneous regions such as cerebrospinal fluid or white matter. These theoretical properties not only justify their selection in the current implementation but also explain the differences observed in experimental metrics such as PSNR, SSIM, and RMSE across the different algorithms.

#### 3.3.4. Metrics of Evaluation

The computational complexity of the employed algorithm is assessed by the execution time and the memory used. The elapsed time is measured in seconds using the tic and toc functions. The memory is measured in gigabytes (GB) using the memory function.

Compression efficiency in this work is defined as the ratio ${∥y∥}_{2}/{∥x∥}_{2}$, where *y* is the vector of sampled transform coefficients and *x* is the original signal. Values close to 1 indicate that the retained coefficients preserve most of the original signal’s energy.

The Root Mean Square Error (RMSE) is used to quantify the difference between the original signal (*x*) and the reconstructed signal (*x1*). It is calculated using the immse function to obtain the Mean Square Error (MSE), and then the square root of this value is taken to get the Root Mean Square Error (RMSE). The formula for MSE is:

(7) $$\mathrm{MSE}=\frac{1}{n}\sum_{i=1}^{n}{\left(x_{i}-x1_{i}\right)}^{2}$$

where $x_{i}$ and $x1_{i}$ are the pixel values of the original and reconstructed images, respectively, and *n* is the total number of pixels. The RMSE is obtained as follows:

(8) $$\mathrm{RMSE}=\sqrt{\mathrm{MSE}}$$

A lower value of RMSE indicates a higher similarity between the images and a minor difference between the predicted and actual values, suggesting a more accurate reconstruction [46].

The Peak Signal-to-Noise Ratio (PSNR) is a metric used to measure the quality of signal reconstruction. It is calculated using the psnr function, which takes as parameters the original signal (*x*) and the reconstructed signal (*x1*). The formula for PSNR is:

(9) $$\mathrm{PSNR}=10\cdot \log_{10}\left(\frac{{\mathrm{MAX}}^{2}}{\mathrm{MSE}}\right)$$

where MAX is the maximum possible pixel value in the image. In the context of medical imaging and Compressed Sensing (CS), a higher PSNR indicates better-reconstructed image quality [47].

#### 3.3.5. Data Loss Metric

In addition to PSNR, RMSE, and SSIM, this study incorporates the Data Loss (DL) metric to quantify the amount of information discarded during the compression and reconstruction process. It is defined as the normalized difference between the original signal *x* and the reconstructed signal $x_{1}$, and is computed as follows:

(10) $$DL=\frac{∥x-x_{1}{∥}_{2}}{{∥x∥}_{2}}\times 100\%$$

where ${∥\cdot ∥}_{2}$ denotes the Euclidean (L2) norm, *x* represents the original image signal, and $x_{1}$ the reconstructed version. The result is expressed as a percentage, indicating the relative loss of information. A lower value of DL corresponds to higher reconstruction fidelity, while a higher value reflects substantial data degradation. This metric offers an intuitive interpretation of signal preservation under various sampling rates and sparsifying transformations.

##### Comparative Metrics

The experimental configurations for data collection for the comparative graphs of RMSE, PSNR, and Data Loss will utilize critical metrics-related variables that vary with time and standardized process iterations. Different sampling rates, ranging from 35% to 75%, will be used in each case.

For calculating the comparative graphs of RMSE, the mean squared error between the original and reconstructed signals will be employed. This value is normalized to facilitate visual comparison in the graphs. Normalization is performed by subtracting the minimum value from the data series and then dividing the result by the range of values. RMSE variants weighted by compression and reconstruction efficiency are also generated, providing different perspectives on the method’s performance. Each algorithm calculates the RMSE by assessing the reconstructed signal after transformation.

The comparative metric PSNR assesses the reconstruction quality in terms of noise. PSNR is expressed in decibels (dB) and is derived from the maximum signal value and the Mean Squared Error (MSE). This value is normalized and used to generate curves showing how reconstruction quality varies with different sampling rates and compression efficiency percentages. Each algorithm computes PSNR by measuring the signal quality after the respective transformations.

Lastly, the Data Loss metric measures the norm of the difference between the original and reconstructed signals, normalized by the norm of the original signal. This value is expressed as a percentage and normalized similarly to RMSE and PSNR. It effectively shows how much information is lost during compression and reconstruction. Data Loss is calculated for each algorithm by measuring the information retained post-transformation.

## 4. Analysis of Results

The implementation of the compressed sensing algorithms was carried out using four different methods: in the first, *DWT*; in the second, *FFT*; in the third, *DCT*; and in the fourth, both *BP* and *DCT*. Each of these algorithms was evaluated on a range of sampling rates, from 35% to 75%, considering evaluation metrics such as compression efficiency, reconstruction quality (*RMSE*, *PSNR*, *SSIM*), computational complexity based on execution time and memory used, and data loss.

**Figure 3:**
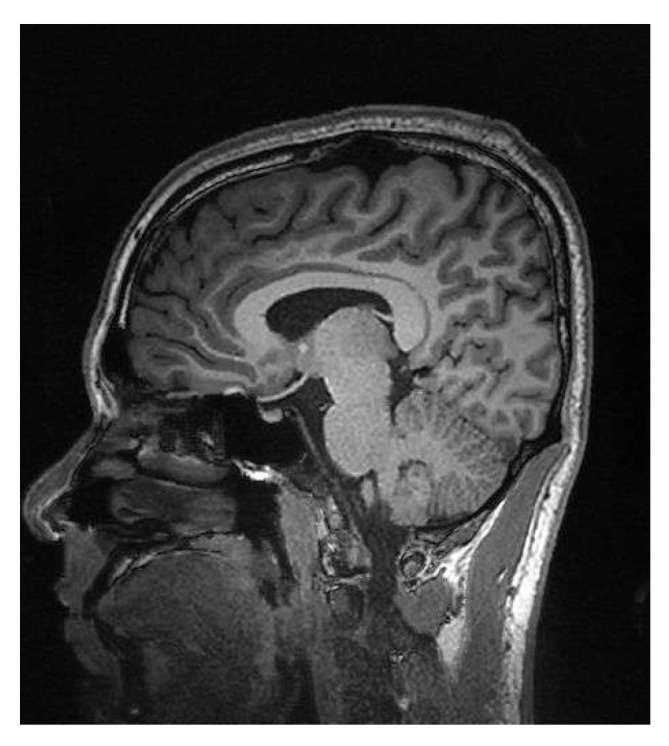
Reference image for the transforms.

Figure 3 shows the original image that was used to obtain the simulated signal by applying the FFT, DCT, or wavelet transforms, thus simulating the signal received from a magnetic resonance imaging (MRI) system. The image was used with the four different proposed algorithms.

**Figure 4:**
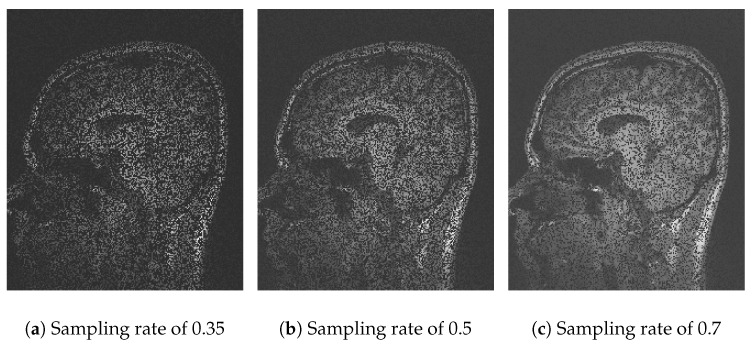
Comparative images for Algorithm 1.

In Figure 4, the performance of Algorithm 1 using the discrete wavelet transform (DWT) is observed. With a sampling rate of 35% (Figure 4a), the reconstruction is poor and lacks information due to the use of one coefficient in the *Waverec* command, which creates reduced square wavelets. By increasing the sampling rate to 50% (Figure 4b) and 70% (Figure 4c), the image quality improves, but spaces without information remain. The results of Algorithm 1 show a *PSNR* of 14.07 dB and an *SSIM* of 0.22 (Table 2). At a sampling rate of 50%, the values improve slightly, reaching a *PSNR* of 15.45 dB and an *SSIM* of 0.32 (Table 3). At a rate of 70%, *PSNR* values of 17.70 dB and an *SSIM* of 0.45 (Table 4) are achieved. The compression efficiency reaches 0.92, indicating that the stored data are closely similar to the original while maintaining a *RMSE* of 13%.

**Figure 5:**
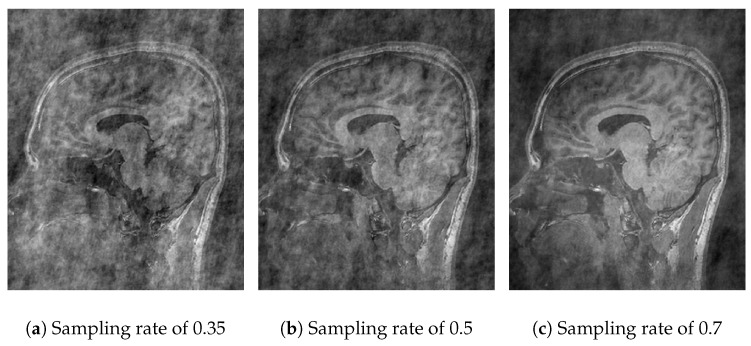
Comparative images for Algorithm 2.

In Figure 5, the performance of Algorithm 2 using the fast Fourier transform (FFT) is analyzed. At a sampling rate of 30% (Figure 5a), an inferior and dark-looking resolution is obtained. Increasing the sampling rate to 50% (Figure 5b) and 70% (Figure 5c), no significant improvement is seen, and the distortions in the form of black spots remain. The results of Algorithm 2 show that at a sampling rate of 30%, the compression efficiency is 0.79, with a *PSNR* of 13.39 dB and an *SSIM* of 0.20 (Table 2). At sampling rates of 50% and 70%, the *PSNR* increases to 18.14 dB and 20.62 dB, respectively, and the *SSIM* to 0.36 and 0.48 (Table 3 and Table 4).

**Figure 6:**
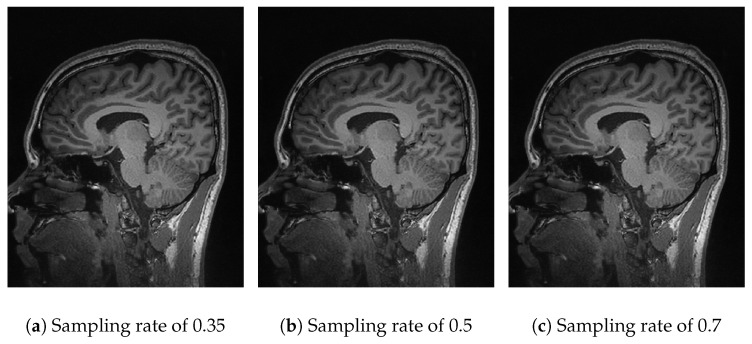
Comparative images for Algorithm 3.

In Figure 6, Algorithm 3, using DCT, is evaluated, showing better compression efficiency and reconstruction quality than the previous transforms. At a sampling rate of 30%, the *PSNR* reaches 44.85 dB, and the *SSIM* is 0.98 (Table 2), reflecting a very accurate reconstruction. At rates of 50% and 70%, the *PSNR* values are 50.52 dB and 57.76 dB, respectively, with *SSIMs* of 0.99 (Table 3 and Table 4), showing better preservation of the original information. The *DCT* is presented as an effective technique for theoretical environments.

While the experimental results demonstrate apparent differences in reconstruction quality, an essential factor for clinical adoption is computational feasibility. The algorithms based on DWT, FFT, and DCT all complete execution in under 3 s, making them suitable for near-real-time applications, such as rapid diagnostic support or preoperative imaging. In contrast, Algorithm 4, which combines basis pursuit and the L_1_ norm with DCT, exhibits execution times exceeding 400 s due to its iterative optimization process. Although this method yields higher-fidelity reconstructions, its computational demand makes it less practical in urgent clinical workflows without hardware acceleration or algorithmic simplification. These trade-offs underscore the importance of striking a balance between reconstruction quality and processing speed when evaluating algorithms for medical applications. Efficient MRI reconstruction must consider not only signal recovery performance but also latency constraints imposed by the clinical environment.

**Figure 7:**
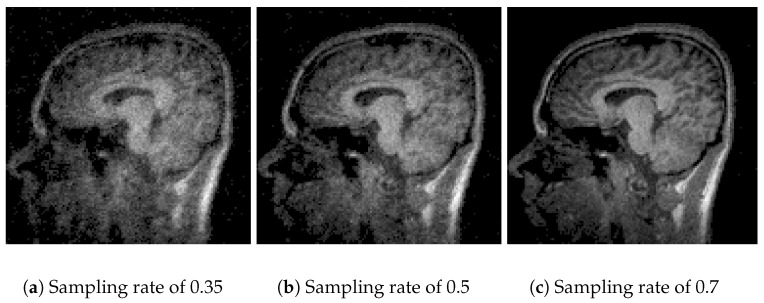
Comparative images for Algorithm 4.

In Figure 7, the performance of Algorithm 4, which combines *basis pursuit* (BP) with *DCT*, is observed. This algorithm shows promising results for real applications. At a sampling rate of 35% (Figure 7a), the results show a *PSNR* of 22.79 dB and an *SSIM* of 0.55 (Table 2), reflecting a reasonable reconstruction quality. At sampling rates of 50% (Figure 7b) and 70% (Figure 7c), the *PSNR* improves significantly to 26.29 dB and 30.27 dB, with *SSIMs* of 0.77 and 0.91 (Table 3 and Table 4). The combination of basis pursuit with *DCT* and the use of a random measurement matrix faithful to the *CS* theory provides efficient compression (0.99) at higher sampling rates (70%) along with high-fidelity reconstruction. Although these results are expected due to the *L*_1_ norm applied to the reconstruction, it is worth noting that the image was resized to 128 × 128 for practical purposes. If a higher resolution were employed, the metrics would tend to rise.

From a theoretical standpoint, each transform offers distinct advantages in MRI reconstruction. DWT preserves structural boundaries through multiscale decomposition, making it suitable for detecting anatomical features. FFT is naturally aligned with k-space acquisition but is sensitive to incoherent sampling. DCT efficiently compresses energy and excels in smooth regions, resulting in superior reconstruction in idealized environments. These transform properties explain the differences in the experimental PSNR, SSIM, and RMSE values.

In terms of computational complexity, DWT, FFT, and DCT can all be executed efficiently in under 3 s, with low memory usage. However, Algorithm 4, which combines basis pursuit and DCT using L_1_ minimization, incurs significantly higher execution times (up to 434 s), limiting its immediate suitability for real-time clinical environments unless optimized or accelerated via parallel processing or hardware-specific implementations.

### Analysis of Graphs of Comparative Metrics

**Figure 8:**
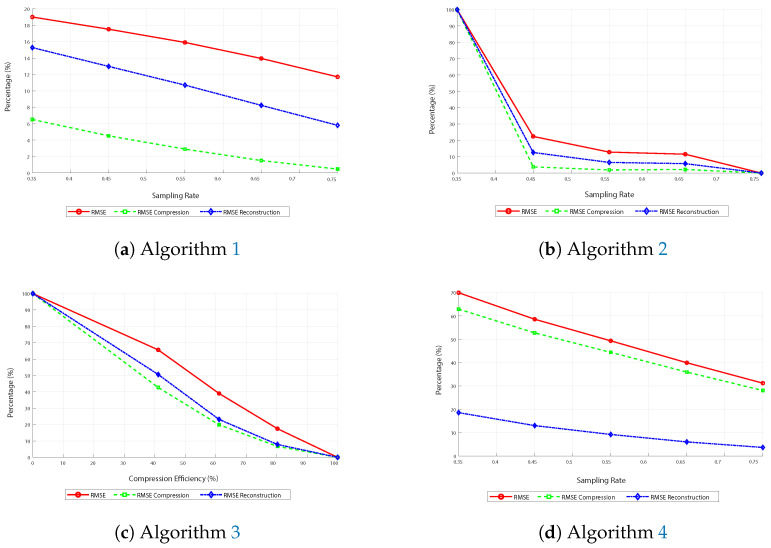
Comparative metrics for RMSE.

The curves generally decrease across all images in Figure 8. This is because the RMSE decreases as the sampling rate increases.

The curves consistently decrease in image (Figure 8a) corresponding to the first algorithm, indicating improved reconstruction and compression efficiency with more data. On the other hand, in image (Figure 8b), corresponding to the FFT-based algorithm, the curves decline sharply until approximately 45% sampling and then descend more gradually. This suggests the method is more effective when at least half of the available subsampling is used.

Similarly, in image (Figure 8c), which shows the performance of the third algorithm based on DCT and subsampling, the RMSE curves exhibit a similar downward trend, albeit with some minor oscillations. This indicates that the algorithm is efficient; however, variations in performance may occur due to the nature of subsampling and reconstruction.

Thus, in the last image (Figure 8d), corresponding to the fourth algorithm that uses DCT, basis pursuit, and the L_1_ norm, the curves also tend to decrease, with a significant reduction in RMSE values as the sampling rate increases. Here, it is also observed that the blue curve differs from the other two, highlighting the algorithm’s superior efficiency in reducing RMSE during reconstruction due to basis pursuit.

**Figure 9:**
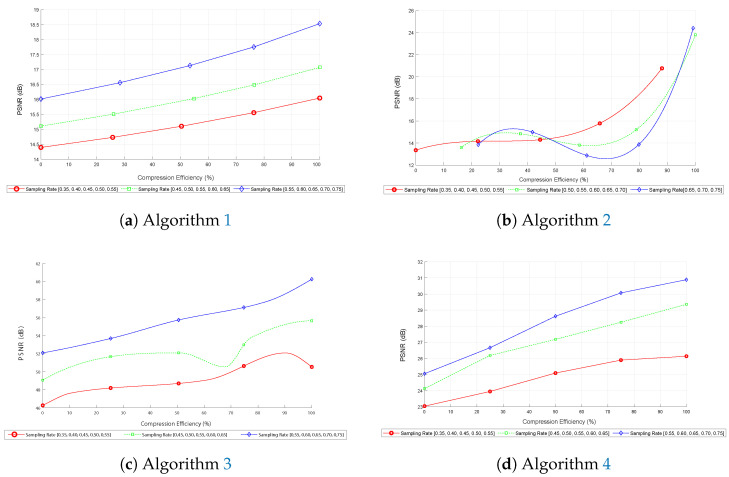
Comparative metrics for PSNR.

Figure 9 presents comparative metrics for PSNR as a function of compression efficiency for different sampling rates.

First, in image (Figure 9a), corresponding to the first algorithm, the curves indicate that PSNR increases consistently with compression efficiency, reaching a maximum of around 19 dB at 100% compression efficiency. This behavior suggests that the algorithm effectively improves reconstruction quality as the sampling rate increases, with the curves maintaining a consistent shape across different sampling rates, indicating robustness under various conditions.

Next, in image (Figure 9b), corresponding to the second algorithm based on the FFT, a similar increase in PSNR is observed, reaching approximately 26 dB. The curves stabilize after a compression efficiency of 50%, suggesting that the algorithm is more effective at higher compression efficiencies. This trend indicates that the FFT algorithm requires fewer data to maintain high reconstruction quality.

Additionally, image (Figure 9c), which shows the performance of the third algorithm based on DCT and subsampling, follows a similar pattern of PSNR increase, reaching values of up to 60 dB. However, minor oscillations are observed in the curves, indicating possible variations in performance due to the nature of subsampling and IDCT reconstruction. This is particularly evident in the green curve within the 45% to 65% range, where the PSNR slightly decreases when compression efficiency is around 70%. These oscillations, though subtle, do not affect the reliability of image reconstruction due to the robustness of DCT in an experimental environment.

Therefore, in graph (Figure 9d), corresponding to the fourth algorithm, that uses DCT, basis pursuit, and the L_1_ norm, the PSNR also tends to increase, reaching a maximum of 32 dB at 100% compression efficiency and sampling rates above 50%. Incorporating basis pursuit and the L_1_ norm mitigates potential information loss, reinforcing the algorithm’s effectiveness in practical applications of compressed sensing.

Overall, the graphs presented in Figure 9 demonstrate that higher sampling rates lead to a better PSNR, indicating improved reconstruction quality. The compression efficiency remains consistent across the curves, providing insights into the performance of each algorithm under different conditions.

**Figure 10:**
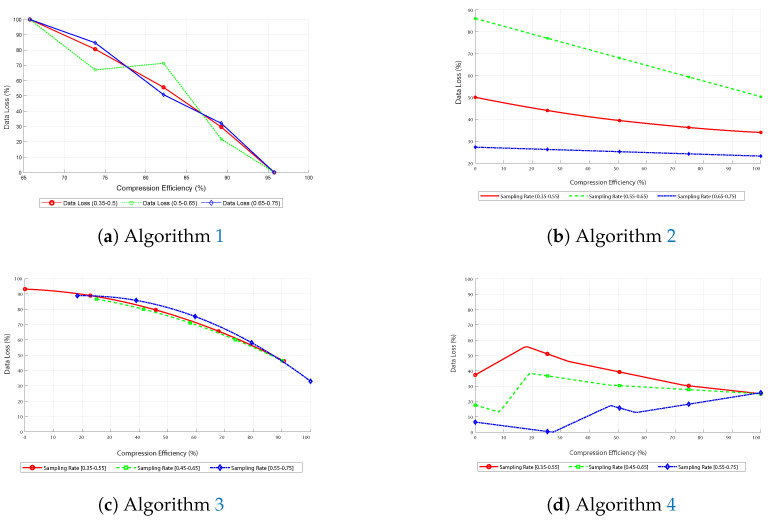
Comparative metrics for data loss.

Each subfigure in Figure 10 illustrates how data loss varies with compression efficiency across different sampling rates.

In image (Figure 10a), the subfigure demonstrates that data loss decreases as compression efficiency increases, reaching a maximum efficiency of 96%. This trend remains consistent across all sampling rates, with only minor variations in specific peaks, indicating the algorithm’s effectiveness.

On the other hand, in graph (Figure 10b), corresponding to Algorithm 2, data loss decreases more smoothly with the increase in compression efficiency, significantly beyond 50%. The curves stabilize more effectively after a gentle decline, indicating an optimal performance point. Due to using FFT, the green curve for a medium sampling rate generated more data loss than even a low sampling rate, attributable to the inherent random factors of CS.

In image (Figure 10c), Algorithm 3 follows a parabolic trend, with a significant reduction in data loss as compression efficiency improves, but with a maximum efficiency of 30%, where the curves converge.

Concluding with image (Figure 10d), this algorithm achieves 100% compression efficiency. However, data loss is inversely proportional to the sampling rate, showing that higher sampling rates result in lower data loss. It is highly effective since it is a reconstruction algorithm that can be applied in real medical scenarios.

## 5. Discussion

While Algorithms 1 and 3 achieved promising performance in simulations, it is emphasized that these methods are not physically consistent with real MRI acquisition. They cannot be directly used for reconstruction from k-space data and serve only as benchmarks for sparsity-based evaluation.

This study has demonstrated that combining traditional inverse reconstruction (FFT) with formal compressed sensing (CS) techniques based on L_1_ minimization can significantly improve image quality and efficiency in MRI simulations. Other transform-based approaches (such as wavelet and DCT) do not correspond to the physical acquisition model of MRI, but were retained to analyze their behavior as sparsity-inducing transforms in controlled environments. The results indicate that higher sampling rates enhance reconstruction quality while maintaining balanced PSNR values and reducing RMSE values across various algorithms, even with rates lower than 50%. These findings align with existing literature on CS, highlighting the balance between sampling rate and reconstruction quality.

Among the four reconstruction methods evaluated, Algorithm 3 (DCT-based reconstruction with subsampling) demonstrated the highest numerical performance across all tested sampling rates, achieving superior values in PSNR (up to 57.76 dB), SSIM (0.99), and RMSE (as low as 0.001). Its computational efficiency also significantly outperformed Algorithm 4, with execution times under 3 s. However, it must be emphasized that Algorithm 3 does not model the physical acquisition process in MRI, as it operates in the k-space domain and lacks an associated sensing matrix. Its superior results highlight the energy compaction properties of the DCT in controlled environments, but they do not qualify it as a clinically applicable CS method. In contrast, Algorithm 4, based on basis pursuit and L_1_-norm minimization, adheres to the compressed sensing framework and reconstructs the image from measurements consistent with the physics of MRI acquisition. Therefore, Algorithm 4 remains the only method in this study with direct translational relevance for real-world MRI applications.

From a practical perspective, optimizing sampling rates with customized algorithms can lead to more efficient MRI procedures, reducing scan times and improving computational costs. The results support that CS techniques can be effectively applied in a hospital environment, enabling high-fidelity reconstructions with the methods studied in this article.

One limitation of this study is the controlled experimental environment, which is based in MATLAB. This limitation should be taken into account when interpreting the results, as it may impact the generalizability of the findings. The study’s focus on specific algorithms may also limit its applicability to other CS techniques. However, it opens new avenues for practical investigations into customized algorithms for more efficient procedures. CS techniques could be effectively applied in a natural hospital environment, as suggested by previous works, such as the study by Azarnia [21], which introduced a PAPR reduction method using CS in OFDM systems, and Wang’s work [48], which combined a new chaotic system with CS and FFT for image encryption. Both studies highlight the versatility and efficacy of CS in various applications within and outside the medical field.

It is also important to acknowledge that the current implementation does not reflect the state of the art in CS-based MRI. Modern reconstruction frameworks such as SPARSE-MRI, SPARSE-SENSE, and L_1_-SPIRiT incorporate parallel acquisition and dynamic sequences, and are optimized for both computational efficiency and clinical fidelity. Moreover, several of these frameworks have already been approved by the FDA, indicating their readiness for routine medical imaging. In contrast, our implementation of basis pursuit via L_1_-MAGIC, while methodologically valid, represents an early-stage formulation. This limitation reduces the direct translational value of the current study and motivates the inclusion of newer CS algorithms for future work.

In light of current trends, it is crucial to acknowledge the growing significance of deep learning and generative artificial intelligence techniques in MRI reconstruction. While the present study focuses on transform-based compressed sensing algorithms, recent advances in data-driven models—including convolutional neural networks, adversarial frameworks, and vision–language architectures—have demonstrated notable improvements in reconstruction speed, image fidelity, and semantic consistency. For example, the work by Zhang et al. (2024) [26] introduces general-purpose vision–language models that integrate imaging features and contextual information to enhance the robustness of reconstruction tasks. Integrating these models into future experimental designs, particularly as hybrid systems combining physical priors with learned representations, represents a compelling direction for further research. Comparative evaluations with such methods will be prioritized in subsequent phases, once suitable GPU-accelerated environments and clinical datasets are available.

Although the results obtained from controlled MRI simulations are promising, the present work does not include clinical testing or real patient data, which is acknowledged as a limitation. The purpose of this study is to provide a computational evaluation platform that can guide the selection or combination of reconstruction methods before their integration into clinical workflows. Future work will address this gap by applying the best-performing configurations, particularly those involving basis pursuit and DCT, to clinical datasets in collaboration with medical imaging professionals, thus enabling validation of diagnostic relevance and real-world applicability.

While this study includes only one L_1_-based compressed sensing method (basis pursuit), future work should incorporate alternative CS reconstruction algorithms using nonconvex regularization. In particular, algorithms with the $L_{0}$ norm (such as greedy methods like orthogonal matching pursuit or hard thresholding pursuit) and those with 0<p<1 (nonconvex minimization) are of high practical relevance. These approaches may offer improved trade-offs between reconstruction quality and computational efficiency and better reflect the theoretical diversity within the CS framework.

## 6. Conclusions

This work presents a comparative evaluation of two MRI-relevant reconstruction methods—FFT and basis pursuit—and two sparsifying transform-based approaches (DWT and DCT), which are included for experimental analysis under simulation. Only the inverse FFT and basis pursuit algorithms are physically consistent with the MRI measurement model y=F(x).

Among the techniques analyzed, the combination of DCT and basis pursuit demonstrated superior reconstruction fidelity under low sampling percentages, achieving an effective balance between image quality and computational efficiency. This supports their suitability for time-constrained MRI scenarios. Additionally, the results showed that even with a sampling percentage below 50%, as defined in Equation (2), acceptable reconstruction quality can be obtained using properly tuned sparse transforms, thereby reducing acquisition time without compromising diagnostic consistency under simulated conditions.

A key contribution of this study lies in the unified comparison framework, which provides consistent benchmarking for sparse reconstruction algorithms. The platform developed in MATLAB serves not only for performance validation but also as a reference for future algorithmic extensions and clinical deployment. This approach addresses the lack of comparative studies under standardized conditions, bridging the gap between theoretical advances and practical implementation.

Although clinical datasets were not included in this phase, the proposed methodology lays the groundwork for translational applications. Future efforts will focus on integrating the most promising configurations—particularly DCT with basis pursuit—into clinical workflows, in collaboration with radiologists and imaging specialists. Furthermore, hybrid methods combining deep learning and sparse models will be explored to enhance reconstruction robustness and semantic consistency, especially for high-resolution or dynamic MRI applications.

Overall, this research reinforces the practical value of CS techniques in modern medical imaging and outlines a clear path toward their integration with next-generation intelligent reconstruction systems.

To strengthen the scientific contribution and clinical relevance of future versions of this work, it is recommended to include more advanced CS algorithms, such as greedy methods with $L_{0}$ regularization (e.g., orthogonal matching pursuit, hard thresholding pursuit) and nonconvex algorithms with 0<p<1. These methods often outperform L_1_-based solvers in both speed and reconstruction quality, providing a more robust comparison against traditional inverse Fourier methods. Their integration would also align the study with current trends in compressed-sensing research and deployment in medical imaging practice.

While this study focused on basis pursuit as a representative L_1_-regularized CS method, future research will incorporate more advanced algorithms. These include greedy methods such as orthogonal matching pursuit (OMP) and hard thresholding pursuit (HTP), as well as nonconvex solvers with 0<p<1. These techniques offer potential advantages in terms of speed and reconstruction quality, aligning with current clinical and computational developments in MRI acceleration.

## Figures and Tables

**Table 1:** Notation and variables used in this article.

Variable	Description
Dimensions	
*K*	Number of non-zero entries in a *K*-sparse vector *s*
*m*	Number of measurements
*n*	Dimension of the original signal $x\in R^{n}$
*p*	Dimension of the measurement variable $y\in R^{p}$
Vectors	
*s*	Sparse vector, $s\in R^{n}$
*x*	Original signal
$x^{\prime }$	Reconstructed signal
*y*	Measurement vector, $y\in R^{p}$
Matrices	
Φ	Projection matrix
Θ	Dictionary matrix Θ=ΦΨ
Ψ	Orthonormal basis (e.g., Fourier, wavelet, Gabor)
$\Psi^{-1}$	Inverse of the orthonormal basis
Norms	
${∥x∥}_{0}$	Pseudo-norm $L_{0}$: number of non-zero elements in *x*
${∥x∥}_{1}$	Norm $L_{1}$: sum of absolute values of *x*’s entries
${∥x∥}_{2}$	Norm $L_{2}$: Euclidean norm of *x*
Transforms	
DCT	Discrete cosine transform
DFT	Discrete Fourier transform
DWT	Discrete wavelet transform
IDCT	Inverse discrete cosine transform
Metrics	
RMSE	Root mean square error
PSNR	Peak signal-to-noise ratio
SSIM	Structural similarity index measure
GB	Memory in gigabytes
[s]	Execution time in seconds
Algorithms	
BP	Basis pursuit
CS	Compressed sensing

**Table 2:** Results with sampling percentage of 35%.

Parameter	Algorithm 1	Algorithm 2	Algorithm 3	Algorithm 4
Execution time [s]	2.02	2.20	2.15	188.51
Memory [GB]	9.79	9.67	9.80	10.88
Compression efficiency	0.60	0.79	0.99	0.99
RMSE	0.19	0.21	0.005	0.07
PSNR [dB]	14.07	13.39	44.85	22.79
SSIM	0.22	0.20	0.98	0.55

**Table 3:** Results with sampling rate of 0.5.

Parameter	Algorithm 1	Algorithm 2	Algorithm 3	Algorithm 4
Execution time [s]	2.58	2.31	2.04	328.63
Memory [GB]	9.79	9.67	9.80	11.67
Compression efficiency	0.77	0.80	1	0.99
RMSE	0.16	0.15	0.002	0.04
PSNR [dB]	15.45	18.14	50.52	26.29
SSIM	0.32	0.36	0.99	0.77

**Table 4:** Results with sampling rate of 0.7.

Parameter	Algorithm 1	Algorithm 2	Algorithm 3	Algorithm 4
Execution time [s]	2.24	2.52	2.18	434.80
Memory [GB]	9.80	9.68	9.80	12.46
Compression efficiency	0.92	0.80	1	0.99
RMSE	0.13	0.12	0.001	0.03
PSNR [dB]	17.70	20.62	57.76	30.27
SSIM	0.45	0.48	0.99	0.91

## Data Availability

The original contributions presented in this study are included in the article. Further inquiries can be directed to the corresponding author.
